# Tyrosine kinase inhibitors: friends or foe in treatment of hepatic fibrosis?

**DOI:** 10.18632/oncotarget.11767

**Published:** 2016-08-31

**Authors:** Kai Qu, Tian Liu, Ting Lin, Xing Zhang, Ruixia Cui, Sinan Liu, Fandi Meng, Jingyao Zhang, Minghui Tai, Yong Wan, Chang Liu

**Affiliations:** ^1^ Department of Hepatobiliary Surgery, The First Affiliated Hospital of Xi'an Jiaotong University, Xi'an, China; ^2^ Department of Hematology, The Second Affiliated Hospital of Xi'an Jiaotong University, Xi'an, China; ^3^ Department of Surgical Intensive Care Unit (SICU), The First Affiliated Hospital of Xi'an Jiaotong University, Xi'an, China; ^4^ Department of Geriatric Surgery, The First Affiliated Hospital of Xi'an Jiaotong University, Xi'an, China; ^5^ Department of Ultrasound, The First Affiliated Hospital of Xi'an Jiaotong University, Xi'an, China

**Keywords:** tyrosine kinase inhibitors, hepatic fibrosis, hepatotoxicity

## Abstract

Aberrant activity of tyrosine kinases has been proved to be associated with multiple diseases including fibrotic diseases. Tyrosine kinases inhibitors (TKIs) might be a novel approach to transform the anti-fibrotic treatment. However, both beneficial and adverse effects are observed by researchers when using these TKIs in either preclinical animal models or patients with hepatic fibrosis. Since hepatotoxicity of TKIs is the leading cause for drug withdrawals thus limits its application in anti-fibrosis, not only efficacy but also safety of TKIs should be paid great concerns. It has been observed in a few studies that TKIs could induce relatively high rate of hepatic biochemical markers elevations and even result in liver failure. Fortunately, several strategies have been adopt to handle with the hepatotoxicity. Accumulating evidences suggest that hepatic stellate cells (HSC) play a pivotal role in hepatic fibrogenesis, so it might be a good option to develop selective TKIs specifically targeting HSCs. The present review will briefly summarize the anti-fibrotic mechanism of TKIs, adverse effects of TKIs as well as the novel developed selective delivery of TKIs.

## INTRODUCTION

**Figure 1:**
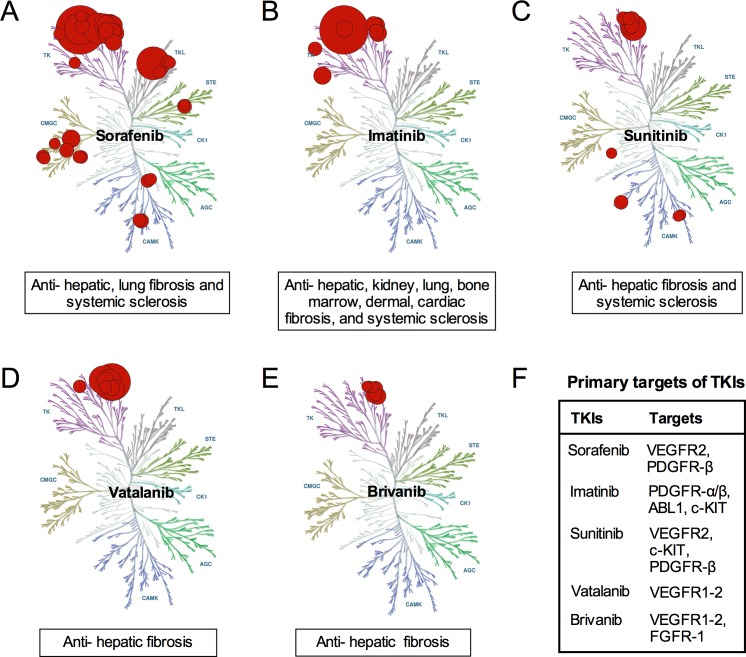
Anti-fibrotic mechanism of several TKIs **A.** Sorafenib exerts several anti-fibrotic effects *via* inhibiting TKs, TKLs, STEs, CMGCs and CAMKs; **B.** Imatinib exerts various anti-fibrosis effects *via* inhibiting of TKs; **C.** Sunitinib exerts anti-fibrosis effects *via* inhibiting TKs and CAMKs; **D.** Vatalanib exert anti-hepatic fibrosis effects *via* inhibiting TKs; **E.** Brivanib exert anti-hepatic fibrosis effects *via* inhibiting TKs; **F.** TKIs exert anti-fibrotic effects by affecting different targets.

Tyrosine kinases (TKs) is a family of tyrosine protein kinases with important functions in regulation of a variety of physiological cell processes. Abnormal TKs activities were found to be associated with non-malignant diseases, including hepatic fibrosis and other fibrotic diseases. Recently, increasing evidence suggested that tyrosine kinases inhibitors (TKIs) seemed to be novel potential drugs for hepatic fibrosis [1]. In preclinical phase, several Food and Drug Administration (FDA)-approved TKIs, such as sorafenib, erlotinib, Imatinib, vatalanib, nilotinib, erotinib and brivanib, exhibited potential anti-fibrosis effects both *in vitro* and *in vivo* (Figure 1). Beneficial effects have been observed by clinicians using above TKIs in some patients with tumors as well as hepatic cirrhosis. However, TKI applications in management of hepatic fibrosis are limited by their hepatotoxicity which has been reported by many clinicians. How to balance the beneficial anti-fibrotic effects and hepatotoxicity of TKIs is a key question and needed to be fully discussed. Although these mentioned controversies have yet remained unanswered, the best advice is to thoroughly understand the mechanisms of anti-fibrosis and hepatotoxicity of TKIs. Hopefully, more details are becoming clear day by day, which have made researchers renew their understandings of TKIs in management of hepatic fibrosis. This review will mainly summarize recent findings and unresolved problems of TKIs in anti-hepatic fibrosis.

## ANTI-FIBROTIC ACTIVITY OF TYROSINE KINASE INHIBITORS: A POTENTIAL NEW THERAPY FOR HEPATIC FIBROSIS

Hepatic fibrosis is defined as the normal liver architecture is replaced by fibrous tissue, scar and regenerative nodules, which leads to liver function loss [2]. Hepatic fibrosis could develop to cirrhosis, hepatocellular carcinoma, or even death. Nowadays, diverse anti-hepatic fibrotic therapies are not seemingly effective from bench to bedside [3]. Accumulating evidence suggested that TKs blocking seems to be a prospective approach to treating hepatic fibrosis, and many animal based preclinical experiments showed that TKIs did bring great benefits to hepatic fibrosis [4, 5]. This should be attributed to its capacity of inhibiting both matrix restructuring and vascular remodeling [6]. In the following section, we will summarize preclinical and clinical evidence for TKIs in management of hepatic fibrosis.

### Anti-fibrotic mechanisms of TKIs in preclinical studies

Grateful thanks to the decades of relevant studies, a numerous biological processes involved in the hepatic fibrogenesis were unveiled. The activation of hepatic stellate cells (HSCs) was considered as a key processes during hepatic fibrogenesis [7–9]. Prior studies have delineated that TKs play an important role in regulating HSC activation [10]. Therefore, targeting TK using inhibitors (TKIs) is considered to be potential approach to inhibit HSC activation and consequently to treat hepatic fibrosis [1]. The mostly investigated TKI which exhibited a high capacity in inhibiting HSC activation is sorafenib. It was found that sorafenib could inhibit proliferation of HSCs by downregulating expression of cyclins and cyclin dependent kinases (CDKs) and prevent ERK, Akt and 70-kDa ribosomal S6 kinase (p70S6K) from phosphorylation [11, 12], [13]. In addition, several other TKIs, such as imatinib [14], vatalanib [15–17], nilotinib [18–22], erlotinib [23, 24] and brivanib [25, 26], were also found to prevent HSC activation, resulting in less collagen deposition.

Portal hypertension is a complication defined as a portal venous pressure gradient exceeding 5 mm which could leads to liver failure even death [27], thus how to deal with portal hypertension never fail to attract attention. Intrahepatic angiogenesis recently is considered to be involved in sinusoidal resistance and portal hypertension, and finally promotes hepatic fibrosis progression. Vascular endothelial growth factor receptor (VEGFR), which belongs to receptor tyrosine kinase, is a key regulator of physiological angiogenesis. It has been clearly investigated that TKIs targeting VEGFRs significantly affected angiogenesis either in tumor or non-malignant. Thabut D et al. reported that sorafenib is associated with suppressing intrahepatic angiogenesis and attenuating hepatic fibrosis [6]. It has been shown that portal pressure and angiogenesis are reduced and no systemic blood pressure fluctuation appeared in sorafenib treated bile duct ligation (BDL) rats [28–30]. Rho kinase activity is crucial for the effect of sorafenib on intrahepatic angiogenesis and portal hypertension [31]. Besides, other TKIs, such as sunitinib, was also showed the ability to reduce portal vein pressure in cirrhotic rats [29].

### Anti-fibrotic activity of TKIs observed in clinical studies

Reduction of portal pressure has been observed in sorafenib treated patients clinically, with a 36% portal venous flow decreasing at least [32]. Similarly, Pinter M *et al*. also reported that a two-week sorafenib treatment exert positive effect on portal hypertension in HCC patients with hepatic fibrosis [33]. Additionally, hepatopulmonary shunt reduction has also been observed in sorafenib treated patients with hepatic cirrhosis, which might greatly improve the prognosis of these patients [34]. Recently, a placebo-controlled randomized clinical trial has been conducted to evaluate the effects of sorafenib on portal pressure in patients with hepatic cirrhosis (NCT01714609).

### TKIs could prevent as well as reverse hepatic fibrosis

To figure out whether TKIs could prevent or reverse hepatic fibrosis, J.T. Stefano et al. conducted a study to evaluate the effects of sorafenib on hepatic fibrosis in an experimental non-alcoholic steatohepatitis (NASH) model rats. Their study demonstrated that sorafenib could prevent the early stages of fibrosis in NASH model rats [35]. Recently, a study by Ikuo Nakamura *et al*. also proved that hepatic fibrosis in mice were attenuated when brivanib was used at the same time [36]. Above results implied that TKIs might be a promising therapeutic strategy in prevention of hepatic fibrosis. Additionally, TKIs had also been found to make effects on reversing hepatic fibrosis. In a more previous study, Neef M et al. reported that early imatinib treatment induced decrease HSCs activity, thus markedly reverse fibrosis in the first three week after BDL [37]. However, their study showed that imatinib make no difference in advanced fibrosis models. In all, above findings suggested that TKIs could prevent as well as reverse hepatic fibrosis, implying TKIs may represent a novel therapeutic approach to treatment of not only HCC but also hepatic fibrosis.

## HEPATOTOXICITY OF TKIS: AN INEVITABLE ISSUE LIMITING THEIR CLINICAL APPLICATION

The leading cause for drug withdrawals is drug toxicity, especially the hepatotoxicity. During the period 1953 to December 2013, 81 medicinal products (18%) that were withdrawn from the market mainly due to hepatotoxicity [38]. An analysis revealed that from 1990 to 2006, 14 of 38 drugs (34.2%) were withdrawn due to hepatotoxicity [39]. As most of TKIs are metabolized by hepatic cytochrome P450 enzyme system [40–42], clinicians should be aware of potential hepatotoxicity with TKIs in patients with liver dysfunction. TKI related hepatotoxicity, including elevation of liver enzymes, liver failure and liver failure induced death, have already been reported in the medical literature [43]. A recent meta-analysis based on 3691 patients received TKIs treatment concluded that hepatotoxicity occurred in 23-40% patients who received TKIs treatment [44]. Additionally, a phase III trial made by Jordi Bruix et al. addressed that the rate of discontinuation of sorafenib for HCC patients after resection or ablation was 50% at a year, far higher than anticipated, which mainly result from adverse effects. Above result indicated that TKIs seemed not tolerable in potentially cured patients [45]. Thus, hepatotoxicity of TKIs is an inevitable issue limiting their application in patients with only hepatic fibrosis. In the following aspect, we summarized that rare but serious and potentially fatal hepatotoxicity of TKIs in clinical trials.

### TKI related hepatotoxicity reported in literature

**Figure 2:**
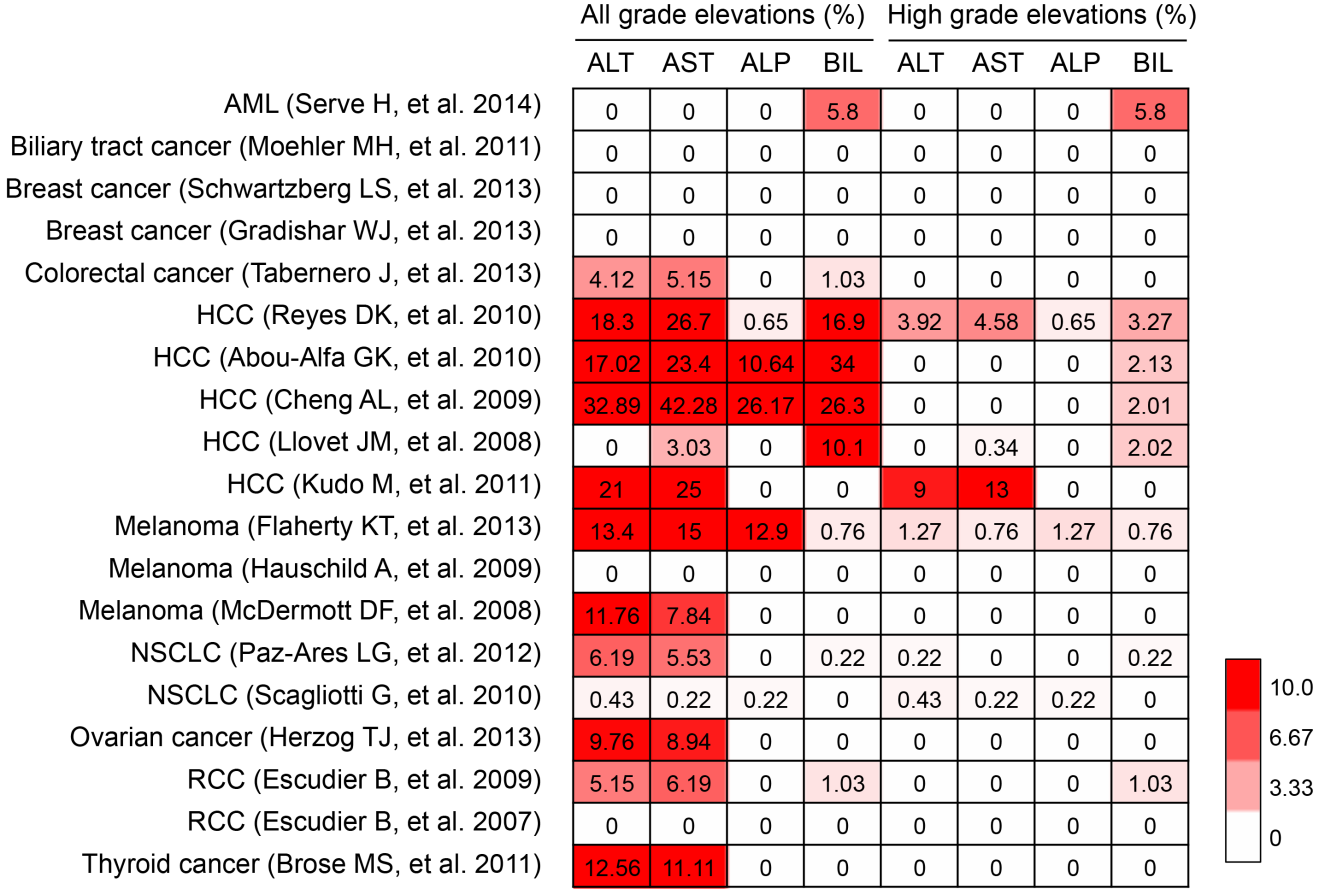
Hepatotoxcity induced by sorafenib AML, acute myeloid leukemia; HCC, hepatocellular carcinoma; NSCLC, non-small cell lung cancer; RCC, renal cell carcinoma. ALT, alanine aminotransferase; AST, aspartate aminotransferase; ALP, alkaline phosphatase; BIL, bilirubin.

Sorafenib is the only TKI which was approved to be used in patients with advanced hepatocellular carcinoma. Whereas in some clinical cases, hepatotoxicity was also reported during administration of sorafenib. Schramm C *et al*. [46] reported that sorafenib could induce liver failure, and Llanos L *et al*. [47] also reported a case of sorafenib-induced severe hepatotoxicity in a 73-year-old man. Hepatotoxicity of sofafenib often manifests as elevations of hepatic biochemical markers, including alanine aminotransferase (ALT), aspartate aminotransferase (AST), alkaline phosphatase (ALP) and bilirubin [43]. Cheng AL et al. even reported that the incidence of AST elevation could be above 40%. To explore the incidence of hepatotoxicity in patients received sorafenib treatment, we therefore conducted a meta-analysis based on 19 clinical trials, including acute myeloid leukemia (AML) (*n* = 1); biliary tract cancer (*n* = 1), breast cancer (*n* = 2), colorectal cancer (*n* = 1), hepatocellular carcinoma (HCC) (*n* = 5), melanoma (*n* = 3), non-small cell lung cancer (NSCLC) (*n* = 2), ovarian cancer (*n* = 1), renal cell carcinoma (RCC) (*n* = 2), thyroid cancer (*n* = 1) [48–66]. Our results revealed that incidences of all-grade elevations of these markers are relatively high in patients treated with sorafenib, especially in HCC patients, who always had a background of chronic liver disease (Figure 2). Moreover, liver failure has also been reported in some cases. P. Ghatalia et al. reported that the overall rate of sorafenib-induced liver failure is 1.5%, suggesting TKI-related hepatotoxicity should be paid close attention.

In addition, other TKIs were also observed to exhibit hepatotoxicity. For instance, Arora, A. K. et al. reported that erlotinib could induce abnormalities of liver function, they also reported a case of erlotinib-induced acute hepatitis [67]. A recent clinical study implied that use of lapatinib in combination with dexamethasone increased the incidence of hepatotoxicity in metastatic breast cancer patients [68]. And another more recent pre-clinical study reported severe and fatal cases of liver injury with lapatinib use [69]. A meta-analysis revealed that pazopanib significantly increased overall risk of hepatotoxicity but not increased risk of fatal hepatotoxicity [70]. Moreover, acute liver failure caused by imatinib and sunitinib have also been observed in some cases [43, 71, 72]. However, there is a lack of evidence about TKI-induced hepatotoxicity in patients with hepatic fibrosis.

It should be pointed out that patients in pre-approval clinical trials are often well-selected, whereas TKIs therapy may increase the likelihood of toxicity and bring down the probability of benefits in less selected patients [73]. Thus, TKI related hepatotoxicity in clinical use need further in-depth discussion.

### Mechanism of TKI related hepatotoxicity

Several hypotheses have been proposed on the mechanism of TKI related hepatotoxicity. It is supposed to be originated from two aspects: one is involved in direct toxicity *via* formation of reactive metabolites (RM) including unstable conjugates, reactive oxygen species (ROS) and other free radicals, another is associated with indirect toxicity *via* secondary immune reactions [74]. It has been demonstrated that various TKIs could generate RM leading to the rise of potential hepatotoxicity risk. The main metabolites of erlotinib, for example, is epoxide and quinone-imine, which result in injury of liver, intestine and lung. Additionally, CYP3A4 enzyme is found to be related to catalysis of liver and intestine RM in several TKIs such as erlotinib and gefitinib [75].

Accumulating evidences suggest that metabolites of TKIs have higher likelihood of causing multi-organs injury. As the liver is the predominant site for bioactivation and detoxification for TKIs, any potential toxicities generating during the process would bring a localized damage. It has been demonstrated in several studies that hepatic metabolisms of TKIs are likely to cause elevation of liver enzymes, liver failure and fatal DILI (drug-induced liver injury) [76]. It has been revealed that the overall risk of developing hepatotoxicity was more than two fold higher in patients with TKIs therapy than control.

The onset of TKIs related hepatotoxicity is highly variable, whereas it has been found that hepatotoxicity usually occurred with the median of seven weeks after TKIs treatment [43]. The majority of patients would present with asymptomatic ALT abnormalities, while seldom would develop to fatal DILI. Despite the fact that fatality from TK inhibitor-induced hepatotoxicity is much rarer compared to hepatotoxic drugs in other classes, it is also very important to point out that some clinical cases develop into unfavorable events including hepatic cirrhosis and even liver failure.

### Prediction of TKI related hepatotoxicity

TKI related hepatotoxicity is often with a variable latency and lacks a significant correlation between drug dose and frequency to severity of hepatotoxicity, which may result from aberrant drug metabolism [77]. It has been pointed out that several factors may affect susceptibility to hepatotoxicity from TKIs, for instance, patients with chronic infection such as hepatitis B or underlying inflammatory liver conditions were indicated to be more vulnerable to liver injury [78]. Additionally, it has been demonstrated that genetic polymorphisms of the major histocompatibility molecules HLA plays an important role in DILI [79]. Some evidence suggested that the HFE rs707889 TT genotype might account for elevation of ALT treating with pazopanib and UGT1A1*28 was found to be associated with elevation of bilirubin [80, 81]. Thus, genetic biomarkers might be a viable approach to assessing risks of DILI.

Moreover, advanced age and female gender contribute to higher risks for DILI, this may due to CYP (cytochrome P450) activity diminishes with ages and CYP plays an important role in metabolism of exogenous agents [82, 83]. It has been shown in a retrospective study that CYP2D6 genotype alone made no difference in higher risk of hepatotoxicity, however, patients harboring the allele *5 and *10 of CYP2D6 gene were classified as intermediate or poor metabolizers and were more vulnerable to recurrent and severe hepatotoxicity with gefitinib treatment [84]. Besides, gefitinib-related hepatotoxicity is more common in Asians than non-Asians, which may also result from CYP polymorphisms among different ethnic groups [85, 86].

### TKIs combination regimen: better treatment than single agent?

Increasing evidence suggested that hepatic fibrosis is a systematic disease with aberrant microenvironments, including abnormal expression HSCs, ECs (liver sinusoidal endothelial cells) and KCs (Kupffer cells) and interaction among them. TKIs combination treatment could simultaneously direct against several targets and therefore achieved superior effects to single agents. It has been demonstrated by Cheng Liu et al. that sorafenib plus GdCl3 significantly inhibited angiogenesis, proinflammatory cytokines, and the interactions among HSCs, SECs and KCs as well as ameliorated the increases in ALT, AST and TBIL in dimethylnitrosamine (DMN)-induced hepatic fibrosis rats compared to either single agent, suggesting TKIs combination regimen is a more potent therapy in hepatic fibrosis [87]. Naoki Yamamoto et al. proved that DFX (deferasirox) in combination with sorafenib could prevent hepatic fibrosis and attenuate adverse effects better than single agent treatment in HCC rats [88]. However, few studies indicated that TKIs combination regimen is better than single agent when treating hepatic fibrosis patients, which warrant further research.

## SELECTIVE DELIVERY OF TKIS: A POTENTIAL STRATEGY FOR REDUCING TKI RELATED HEPATOTOXICITY

Overwhelming evidences suggest that hepatic stellate cells (HSC) play a pivotal role in hepatic fibrogenesis [10, 89]. Activation of HSC results in wound healing reaction and formation of myofibroblasts. HSC is a major source of the production of various profibrotic cytokines as well as extracellular matrix proteins. Since tyrosine kinase-regulated pathways are associated with almost every cell type of the body, TKIs in non-diseased organs may exert serious adverse effects. Thus, it seems that selective TKIs specifically targeting HSCs may be a good option to improve anti-fibrotic efficacy *via* increasing local drug levels in HSC and decreasing drug levels elsewhere. To optimize the therapy of hepatic fibrosis, targeting to HSC has been explored for years. And here are several approaches to HSC targeted therapy have been developed [90, 91].

### Selective protein carriers

It has been demonstrated that receptors such as PDGF-β receptor are highly upregulated on activated HSC, thus using albumin-based carriers that bind to these receptors could be a novel approach. In experimental animal models of hepatic fibrosis, it has been revealed that these carriers open the opportunity to delivery majority of anti-fibrotic compounds to HSC. In the past few years, drugs like kinase inhibitors affecting PDGF-β signaling pathway were successfully delivered to these cells in experimental animals [91]. Homing device of mannose 6-phosphate (M6P) is also a significant method to deliver drugs to HSC. Researchers have combined M6P with a RGD homing device, and it has been proved that M6P-RGD construction is more effective in inhibiting hepatic fibrosis than M6P or RGD alone, suggesting that M6P-RGD is also a good approach to target HSC selectively [92]. PDGFR-β is one of the most important pro-fibrotic TKs in hepatic fibrosis signals. This pathway can be inhibit by imatinib, however, this kind of drugs would bring severe side effect. PAP19 is an imatinib-like drug that inhibits the PDGFR-β. Gonzalo T et al. have demonstrated that PAP19 reduce fibrotic markers in HSC and target HSC specifically in a rat model of hepatic fibrosis via the M6PHSA carrier [93].

### Other nanoparticle-based drug vehicles

Nanoparticles are frequently defined as particles sized from one up to 500 nm. It is widely known that metal-based nanotherapeutics have brought about a series of achievements such as computerized tomography [94]. Size and functionalization of nanoparticles decide its distribution in different organs. Therefore, organic nanoparticles such as lipid could be considered as a novel nanocarriers. Chai et al. also applied RGD peptide-labeled liposomes to deliver oxymatrine (OM) specifically into HSCs. OM is an herbal medicinal product that could bring benefits to hepatic fibrosis in rats. And in this study, researchers formed an OM-liposomes compound and coupled the RGD peptides and the OM-liposomes afterwards. This compound showed excellent ability of reducing collagen deposition and hepatic fibrosis related genes expression, suggesting liposomes could be a wonderful candidate for delivery of antifibrotic drugs [95].

## PERSPECTIVES

It cannot be denied that TKIs make a great contribution to transforming many cancers from a death sentence to a chronic illness. Accumulating evidences have demonstrated that multitargeted TKIs are capable of bringing benefit not only to malignant tumor but also fibrotic disease. During fibrogenesis, many intracellular signaling pathways are inappropriately activated, in which TKs plays a significant role. Thus, TKIs seems to be a novel potential treatment for fibrotic disease. And it has been identified in multiple preclinical studies that TKIs could either prevent or reverse hepatic fibrosis. As a result, based on the advantages of TKIs, targeted therapy might become major approaches for treating hepatic fibrosis in future.

However, it should never fail to point out that toxicity of TKIs is a serious problem and hence hinter the TKIs application. In spite of the common adverse effects of TKIs including rash, gastrointestinal symptoms, fatigue, edema, and neurological symptoms are mild and tolerable for patients, some severe effects like liver function impairment and even acute liver failure have been reported clinically. Generally, most existing TKIs have no specific selectivity between the normal cells and the tumor or fibrotic cells, implying potential systematic adverse effect. Increasing evidence from preclinical studies implied that discontinuation of TKIs was resulted from intolerable side effects thus limited its application in patients with only hepatic fibrosis. Therefore, the adverse effects of TKIs especially hepatotoxicity is the problem demanding most concern.

Developing TKIs that target only specific cells should be paid close attention. As we all know that TKIs withdrawals resulting from hepatotoxicity are quite common, TKIs with cell selectivity may decrease the risk of toxicity. Moreover, TKIs might exert excessive economic pressure on patients, which also would put sand on their applications for hepatic fibrosis. Given together, TKIs, as discussed in this review, are novel potential drugs for hepatic fibrosis, but still existing lots of problems to be handled with. In the near future, clinical application of TKIs on hepatic fibrosis should turn out to be not only an efficient but also safety treatment.

## References

[1] Heldin CH (2014). Targeting the PDGF signaling pathway in the treatment of non-malignant diseases. Journal of neuroimmune pharmacology.

[2] Hernandez-Gea V, Friedman SL (2011). Pathogenesis of liver fibrosis. Annual review of pathology.

[3] Friedman SL (2003). Liver fibrosis -- from bench to bedside. Journal of hepatology.

[4] Rossler J, Geoerger B, Taylor M, Vassal G (2008). Small molecule tyrosine kinase inhibitors: potential role in pediatric malignant solid tumors. Current cancer drug targets.

[5] Grimminger F, Schermuly RT, Ghofrani HA (2010). Targeting non-malignant disorders with tyrosine kinase inhibitors. Nature reviews Drug discovery.

[6] Thabut D, Routray C, Lomberk G, Shergill U, Glaser K, Huebert R, Patel L, Masyuk T, Blechacz B, Vercnocke A, Ritman E, Ehman R, Urrutia R, Shah V (2011). Complementary vascular and matrix regulatory pathways underlie the beneficial mechanism of action of sorafenib in liver fibrosis. Hepatology.

[7] Pinzani M, Marra F (2001). Cytokine receptors and signaling in hepatic stellate cells. Seminars in liver disease.

[8] Kawada N (2004). Molecular mechanism of stellate cell activation and therapeutic strategy for liver fibrosis. Comparative hepatology.

[9] Yang CQ, Yang L, Yang WZ, Zhang Z, Zhang H, Chang YZ, Yuan M, Chen XM (2008). [Mechanism of hepatic stellate cell migration during liver fibrosis]. Zhonghua yi xue za zhi.

[10] Friedman SL (2008). Mechanisms of hepatic fibrogenesis. Gastroenterology.

[11] Plastaras JP, Kim SH, Liu YY, Dicker DT, Dorsey JF, McDonough J, Cerniglia G, Rajendran RR, Gupta A, Rustgi AK, Diehl JA, Smith CD, Flaherty KT, El-Deiry WS (2007). Cell cycle dependent and schedule-dependent antitumor effects of sorafenib combined with radiation. Cancer research.

[12] Tomizawa M, Shinozaki F, Sugiyama T, Yamamoto S, Sueishi M, Yoshida T (2010). Sorafenib suppresses the cell cycle and induces the apoptosis of hepatocellular carcinoma cell lines in serum-free media. Experimental and therapeutic medicine.

[13] Wang Y, Gao J, Zhang D, Zhang J, Ma J, Jiang H (2010). New insights into the antifibrotic effects of sorafenib on hepatic stellate cells and liver fibrosis. Journal of hepatology.

[14] Yoshiji H, Noguchi R, Kuriyama S, Ikenaka Y, Yoshii J, Yanase K, Namisaki T, Kitade M, Masaki T, Fukui H (2005). Imatinib mesylate (STI-571) attenuates liver fibrosis development in rats. American journal of physiology Gastrointestinal and liver physiology.

[15] Wood JM, Bold G, Buchdunger E, Cozens R, Ferrari S, Frei J, Hofmann F, Mestan J, Mett H, O'Reilly T, Persohn E, Rosel J, Schnell C (2000). PTK787/ZK 222584, a novel and potent inhibitor of vascular endothelial growth factor receptor tyrosine kinases, impairs vascular endothelial growth factor-induced responses and tumor growth after oral administration. Cancer research.

[16] Liu Y, Lui EL, Friedman SL, Li L, Ye T, Chen Y, Poon RT, Wo J, Kok TW, Fan ST (2009). PTK787/ZK22258 attenuates stellate cell activation and hepatic fibrosis in vivo by inhibiting VEGF signaling. Laboratory investigation; a journal of technical methods and pathology.

[17] Liu Y, Wen XM, Lui EL, Friedman SL, Cui W, Ho NP, Li L, Ye T, Fan ST, Zhang H (2009). Therapeutic targeting of the PDGF and TGF-beta-signaling pathways in hepatic stellate cells by PTK787/ZK22258. Laboratory investigation; a journal of technical methods and pathology.

[18] Shaker ME, Ghani A, Shiha GE, Ibrahim TM, Mehal WZ (2013). Nilotinib induces apoptosis and autophagic cell death of activated hepatic stellate cells via inhibition of histone deacetylases. Biochimica et biophysica acta.

[19] Shaker ME, Salem HA, Shiha GE, Ibrahim TM (2011). Nilotinib counteracts thioacetamide-induced hepatic oxidative stress and attenuates liver fibrosis progression. Fundamental & clinical pharmacology.

[20] Shaker ME, Shiha GE, Ibrahim TM (2011). Comparison of early treatment with low doses of nilotinib, imatinib and a clinically relevant dose of silymarin in thioacetamide-induced liver fibrosis. European journal of pharmacology.

[21] Shaker ME, Zalata KR, Mehal WZ, Shiha GE, Ibrahim TM (2011). Comparison of imatinib, nilotinib and silymarin in the treatment of carbon tetrachloride-induced hepatic oxidative stress, injury and fibrosis. Toxicology and applied pharmacology.

[22] Shiha GE, Abu-Elsaad NM, Zalata KR, Ibrahim TM (2014). Tracking anti-fibrotic pathways of nilotinib and imatinib in experimentally induced liver fibrosis: an insight. Clinical and experimental pharmacology & physiology.

[23] Bach JP, Kiessling AM, Kleinhans A, Faoro C, Gorg C, Riera-Knorrenschild J, Schwella N, Wagner HJ, Neubauer A (2006). Pulmonary fibrosis in a patient treated with erlotinib. Onkologie.

[24] Fuchs BC, Hoshida Y, Fujii T, Wei L, Yamada S, Lauwers GY, McGinn CM, DePeralta DK, Chen X, Kuroda T, Lanuti M, Schmitt AD, Gupta S (2014). Epidermal growth factor receptor inhibition attenuates liver fibrosis and development of hepatocellular carcinoma. Hepatology.

[25] Lin HC, Huang YT, Yang YY, Lee PC, Hwang LH, Lee WP, Kuo YJ, Lee KC, Hsieh YC, Liu RS (2014). Beneficial effects of dual vascular endothelial growth factor receptor/fibroblast growth factor receptor inhibitor brivanib alaninate in cirrhotic portal hypertensive rats. Journal of gastroenterology and hepatology.

[26] Yang YY, Liu RS, Lee PC, Yeh YC, Huang YT, Lee WP, Lee KC, Hsieh YC, Lee FY, Tan TW, Lin HC (2014). Anti-VEGFR agents ameliorate hepatic venous dysregulation/microcirculatory dysfunction, splanchnic venous pooling and ascites of NASH-cirrhotic rat. Liver international.

[27] Lee JS, Kim JH (2007). [The role of activated hepatic stellate cells in liver fibrosis, portal hypertension and cancer angiogenesis]. The Korean journal of hepatology.

[28] Mejias M, Garcia-Pras E, Tiani C, Miquel R, Bosch J, Fernandez M (2009). Beneficial effects of sorafenib on splanchnic, intrahepatic, and portocollateral circulations in portal hypertensive and cirrhotic rats. Hepatology.

[29] Tugues S, Fernandez-Varo G, Munoz-Luque J, Ros J, Arroyo V, Rodes J, Friedman SL, Carmeliet P, Jimenez W, Morales-Ruiz M (2007). Antiangiogenic treatment with sunitinib ameliorates inflammatory infiltrate, fibrosis, and portal pressure in cirrhotic rats. Hepatology.

[30] Rosmorduc O (2010). Antiangiogenic therapies in portal hypertension: a breakthrough in hepatology. Gastroenterologie clinique et biologique.

[31] Hennenberg M, Trebicka J, Stark C, Kohistani AZ, Heller J, Sauerbruch T (2009). Sorafenib targets dysregulated Rho kinase expression and portal hypertension in rats with secondary biliary cirrhosis. British journal of pharmacology.

[32] Coriat R, Gouya H, Mir O, Ropert S, Vignaux O, Chaussade S, Sogni P, Pol S, Blanchet B, Legmann P, Goldwasser F (2011). Reversible decrease of portal venous flow in cirrhotic patients: a positive side effect of sorafenib. PloS one.

[33] Pinter M, Sieghart W, Reiberger T, Rohr-Udilova N, Ferlitsch A, Peck-Radosavljevic M (2012). The effects of sorafenib on the portal hypertensive syndrome in patients with liver cirrhosis and hepatocellular carcinoma--a pilot study. Alimentary pharmacology & therapeutics.

[34] Theysohn JM, Schlaak JF, Muller S, Ertle J, Schlosser TW, Bockisch A, Lauenstein TC (2012). Selective internal radiation therapy of hepatocellular carcinoma: potential hepatopulmonary shunt reduction after sorafenib administration. Journal of vascular and interventional radiology.

[35] Stefano JT, Pereira IV, Torres MM, Bida PM, Coelho AM, Xerfan MP, Cogliati B, Barbeiro DF, Mazo DF, Kubrusly MS, D'Albuquerque LA, Souza HP, Carrilho FJ, Oliveira CP (2015). Sorafenib prevents liver fibrosis in a non-alcoholic steatohepatitis (NASH) rodent model. Brazilian journal of medical and biological research.

[36] Nakamura I, Zakharia K, Banini BA, Mikhail DS, Kim TH, Yang JD, Moser CD, Shaleh HM, Thornburgh SR, Walters I, Roberts LR (2014). Brivanib attenuates hepatic fibrosis in vivo and stellate cell activation in vitro by inhibition of FGF, VEGF and PDGF signaling. PloS one.

[37] Neef M, Ledermann M, Saegesser H, Schneider V, Widmer N, Decosterd LA, Rochat B, Reichen J (2006). Oral imatinib treatment reduces early fibrogenesis but does not prevent progression in the long term. Journal of hepatology.

[38] Onakpoya IJ, Heneghan CJ, Aronson JK (2016). Post-marketing withdrawal of 462 medicinal products because of adverse drug reactions: a systematic review of the world literature. BMC medicine.

[39] Shah RR (2006). Can pharmacogenetics help rescue drugs withdrawn from the market?. Pharmacogenomics.

[40] Lathia C, Lettieri J, Cihon F, Gallentine M, Radtke M, Sundaresan P (2006). Lack of effect of ketoconazole-mediated CYP3A inhibition on sorafenib clinical pharmacokinetics. Cancer chemotherapy and pharmacology.

[41] Peer CJ, Sissung TM, Kim A, Jain L, Woo S, Gardner ER, Kirkland CT, Troutman SM, English BC, Richardson ED, Federspiel J, Venzon D, Dahut W (2012). Sorafenib is an inhibitor of UGT1A1 but is metabolized by UGT1A9: implications of genetic variants on pharmacokinetics and hyperbilirubinemia. Clinical cancer research.

[42] Druker BJ (2003). Imatinib mesylate in the treatment of chronic myeloid leukaemia. Expert opinion on pharmacotherapy.

[43] Shah RR, Morganroth J, Shah DR (2013). Hepatotoxicity of tyrosine kinase inhibitors: clinical and regulatory perspectives. Drug Saf.

[44] Iacovelli R, Palazzo A, Procopio G, Santoni M, Trenta P, De Benedetto A, Mezi S, Cortesi E (2014). Incidence and relative risk of hepatic toxicity in patients treated with anti-angiogenic tyrosine kinase inhibitors for malignancy. British journal of clinical pharmacology.

[45] Bruix J, Takayama T, Mazzaferro V, Chau GY, Yang J, Kudo M, Cai J, Poon RT, Han KH, Tak WY, Lee HC, Song T, Roayaie S (2015). Adjuvant sorafenib for hepatocellular carcinoma after resection or ablation (STORM): a phase 3, randomised, double-blind, placebo-controlled trial. The Lancet Oncology.

[46] Schramm C, Schuch G, Lohse AW (2008). Sorafenib-induced liver failure. The American journal of gastroenterology.

[47] Llanos L, Bellot P, Zapater P, Perez-Mateo M, Such J (2009). Acute hepatitis in a patient with cirrhosis and hepatocellular carcinoma treated with sorafenib. The American journal of gastroenterology.

[48] Tabernero J, Garcia-Carbonero R, Cassidy J, Sobrero A, Van Cutsem E, Koehne C-H, Tejpar S, Gladkov O, Davidenko I, Salazar R, Vladimirova L, Cheporov S, Burdaeva O (2013). Sorafenib in Combination with Oxaliplatin, Leucovorin, and Fluorouracil (Modified FOLFOX6) as First-line Treatment of Metastatic Colorectal Cancer: The RESPECT Trial. Clinical Cancer Research.

[49] Serve H, Krug U, Wagner R, Sauerland MC, Heinecke A, Brunnberg U, Schaich M, Ottmann O, Duyster J, Wandt H, Fischer T, Giagounidis A, Neubauer A (2013). Sorafenib in Combination With Intensive Chemotherapy in Elderly Patients With Acute Myeloid Leukemia: Results From a Randomized, Placebo-Controlled Trial. Journal of Clinical Oncology.

[50] Schwartzberg LS, Tauer KW, Hermann RC, Makari-Judson G, Isaacs C, Beck JT, Kaklamani V, Stepanski EJ, Rugo HS, Wang W, Bell-McGuinn K, Kirshner JJ, Eisenberg P (2013). Sorafenib or Placebo with Either Gemcitabine or Capecitabine in Patients with HER-2-Negative Advanced Breast Cancer That Progressed during or after Bevacizumab. Clinical Cancer Research.

[51] Scagliotti G, Novello S, von Pawel J, Reck M, Pereira JR, Thomas M, Abrao Miziara JE, Balint B, De Marinis F, Keller A, Aren O, Csollak M, Albert I (2010). Phase III Study of Carboplatin and Paclitaxel Alone or With Sorafenib in Advanced Non Small-Cell Lung Cancer. Journal of Clinical Oncology.

[52] Reyes DK (2010). Phase II trial of sorafenib combined with doxorubicin-eluting bead transarterial chemoembolization (DEB-TACE) for patients with unresectable hepatocellular carcinoma (HCC): interim safety and efficacy analysis. Gastrointestinal cancer symposium.

[53] Paz-Ares LG, Biesma B, Heigener D, von Pawel J, Eisen T, Bennouna J, Zhang L, Liao M, Sun Y, Gans S, Syrigos K, Le Marie E, Gottfried M (2012). Phase III, Randomized, Double-Blind, Placebo-Controlled Trial of Gemcitabine/Cisplatin Alone or With Sorafenib for the First-Line Treatment of Advanced, Nonsquamous Non-Small-Cell Lung Cancer. Journal of Clinical Oncology.

[54] Moehler MH, Schimanski C, Kanzler S (2011). A randomized, double-blind, multicenter phase II AIO trial with gemcitabine plus sorafenib versus gemcitabine plus placebo in patients with chemotherapy-naive advanced or metastatic biliary tract cancer: first safety and efficacy data. Journal of clinical oncology.

[55] McDermott DF, Sosman JA, Gonzalez R, Hodi FS, Linette GP, Richards J, Jakub W, Beeram M, Tarantolo S, Agarwala S, Frenette G, Puzanov I, Cranmer L (2008). Double-blind randomized phase II study of the combination of sorafenib and dacarbazine in patients with advanced melanoma: A report from the 11715 study group. Journal of Clinical Oncology.

[56] Llovet JM, Ricci S, Mazzaferro V, Hilgard P, Gane E, Blanc J-F, Cosme de Oliveira A, Santoro A, Raoul J-L, Forner A, Schwartz M, Porta C, Zeuzem S (2008). Sorafenib in advanced hepatocellular carcinoma. New England Journal of Medicine.

[57] Kudo M, Imanaka K, Chida N, Nakachi K, Tak W-Y, Takayama T, Yoon J-H, Hori T, Kumada H, Hayashi N, Kaneko S, Tsubouchi H, Suh DJ (2011). Phase III study of sorafenib after transarterial chemoembolisation in Japanese and Korean patients with unresectable hepatocellular carcinoma. European Journal of Cancer.

[58] Herzog TJ, Scambia G, Kim B-G, Lhomme C, Markowska J, Ray-Coquard I, Sehouli J, Colombo N, Shan M, Petrenciuc O, Oza A (2013). A randomized phase II trial of maintenance therapy with Sorafenib in front-line ovarian carcinoma. Gynecologic Oncology.

[59] Hauschild A, Agarwala SS, Trefzer U, Hogg D, Robert C, Hersey P, Eggermont A, Grabbe S, Gonzalez R, Gille J, Peschel C, Schadendorf D, Garbe C (2009). Results of a Phase III, Randomized, Placebo-Controlled Study of Sorafenib in Combination With Carboplatin and Paclitaxel As Second-Line Treatment in Patients With Unresectable Stage III or Stage IV Melanoma. Journal of Clinical Oncology.

[60] Gradishar WJ, Kaklamani V, Sahoo TP, Lokanatha D, Raina V, Bondarde S, Jain M, Ro SK, Lokker NA, Schwartzberg L (2013). A double-blind, randomised, placebo-controlled, phase 2b study evaluating sorafenib in combination with paclitaxel as a first-line therapy in patients with HER2-negative advanced breast cancer. European Journal of Cancer.

[61] Flaherty KT, Lee SJ, Zhao F, Schuchter LM, Flaherty L, Kefford R, Atkins MB, Leming P, Kirkwood JM (2013). Phase III Trial of Carboplatin and Paclitaxel With or Without Sorafenib in Metastatic Melanoma. Journal of Clinical Oncology.

[62] Escudier B, Szczylik C, Hutson TE, Demkow T, Staehler M, Rolland F, Negrier S, Laferriere N, Scheuring UJ, Cella D, Shah S, Bukowski RM (2009). Randomized Phase II Trial of First-Line Treatment With Sorafenib Versus Interferon Alfa-2a in Patients With Metastatic Renal Cell Carcinoma. Journal of Clinical Oncology.

[63] Escudier B, Eisen T, Stadler WM, Szczylik C, Oudard S, Siebels M, Negrier S, Chevreau C, Solska E, Desai AA, Rolland F, Demkow T, Hutson TE (2007). Sorafenib in advanced clear-cell renal-cell carcinoma. New England Journal of Medicine.

[64] Cheng A-L, Kang Y-K, Chen Z, Tsao C-J, Qin S, Kim JS, Luo R, Feng J, Ye S, Yang T-S, Xu J, Sun Y, Liang H (2009). Efficacy and safety of sorafenib in patients in the Asia-Pacific region with advanced hepatocellular carcinoma: a phase III randomised, double-blind, placebo-controlled trial. Lancet Oncology.

[65] Brose MS, Nutting CM, Sherman SI, Shong YK, Smit JWA, Reike G, Chung J, Kalmus J, Kappeler C, Schlumberger M (2011). Rationale and design of decision: a double-blind, randomized, placebo-controlled phase III trial evaluating the efficacy and safety of sorafenib in patients with locally advanced or metastatic radioactive iodine (RAI)-refractory, differentiated thyroid cancer. Bmc Cancer.

[66] Abou-Alfa GK, Johnson P, Knox JJ, Capanu M, Davidenko I, Lacava J, Leung T, Gansukh B, Saltz LB (2010). Doxorubicin Plus Sorafenib vs Doxorubicin Alone in Patients With Advanced Hepatocellular Carcinoma A Randomized Trial. Jama-Journal of the American Medical Association.

[67] Arora AK (2011). Erlotinib-induced Hepatotoxicity-Clinical Presentation and Successful Management: A Case Report. Journal of clinical and experimental hepatology.

[68] Moy B, Rappold E, Williams L, Kelly T, Nicolodi L, Maltzman J, Goss P (2009). Hepatobiliary abnormalities in patients with metastatic cancer treated with lapatinib.

[69] Cristofanilli M, Johnston SR, Manikhas A, Gomez HL, Gladkov O, Shao Z, Safina S, Blackwell KL, Alvarez RH, Rubin SD, Ranganathan S, Redhu S, Trudeau ME (2013). A randomized phase II study of lapatinib + pazopanib versus lapatinib in patients with HER2+ inflammatory breast cancer. Breast cancer research and treatment.

[70] Kapadia S, Hapani S, Choueiri TK, Wu S (2013). Risk of liver toxicity with the angiogenesis inhibitor pazopanib in cancer patients. Acta oncologica (Stockholm, Sweden).

[71] Cross TJ, Bagot C, Portmann B, Wendon J, Gillett D (2006). Imatinib mesylate as a cause of acute liver failure. American journal of hematology.

[72] Tonyali O, Coskun U, Yildiz R, Karakan T, Demirci U, Akyurek N, Benekli M, Buyukberber S (2010). Imatinib mesylate-induced acute liver failure in a patient with gastrointestinal stromal tumors. Medical oncology.

[73] Seruga B, Sterling L, Wang L, Tannock IF (2011). Reporting of serious adverse drug reactions of targeted anticancer agents in pivotal phase III clinical trials. Journal of clinical oncology.

[74] Walgren JL, Mitchell MD, Thompson DC (2005). Role of metabolism in drug-induced idiosyncratic hepatotoxicity. Critical reviews in toxicology.

[75] Teo YL, Ho HK, Chan A (2015). Formation of reactive metabolites and management of tyrosine kinase inhibitor-induced hepatotoxicity: a literature review. Expert opinion on drug metabolism & toxicology.

[76] Lammert C, Bjornsson E, Niklasson A, Chalasani N (2010). Oral medications with significant hepatic metabolism at higher risk for hepatic adverse events. Hepatology.

[77] Gunawan BK, Kaplowitz N (2007). Mechanisms of drug-induced liver disease. Clinics in liver disease.

[78] Levy M (1997). Role of viral infections in the induction of adverse drug reactions. Drug Saf.

[79] Spraggs CF, Xu CF, Hunt CM (2013). Genetic characterization to improve interpretation and clinical management of hepatotoxicity caused by tyrosine kinase inhibitors. Pharmacogenomics.

[80] Xu CF, Reck BH, Goodman VL, Xue Z, Huang L, Barnes MR, Koshy B, Spraggs CF, Mooser VE, Cardon LR, Pandite LN (2011). Association of the hemochromatosis gene with pazopanib-induced transaminase elevation in renal cell carcinoma. Journal of hepatology.

[81] Xu CF, Reck BH, Xue Z, Huang L, Baker KL, Chen M, Chen EP, Ellens HE, Mooser VE, Cardon LR, Spraggs CF, Pandite L (2010). Pazopanib-induced hyperbilirubinemia is associated with Gilbert's syndrome UGT1A1 polymorphism. British journal of cancer.

[82] Chalasani N, Fontana RJ, Bonkovsky HL, Watkins PB, Davern T, Serrano J, Yang H, Rochon J (2008). Causes, clinical features, and outcomes from a prospective study of drug-induced liver injury in the United States. Gastroenterology.

[83] Benedetti MS, Whomsley R, Canning M (2007). Drug metabolism in the paediatric population and in the elderly. Drug discovery today.

[84] Sugiyama E, Umemura S, Nomura S, Kirita K, Matsumoto S, Yoh K, Niho S, Ohmatsu H, Tsuboi M, Ohe Y, Goto K (2015). Impact of single nucleotide polymorphisms on severe hepatotoxicity induced by EGFR tyrosine kinase inhibitors in patients with non-small cell lung cancer harboring EGFR mutations. Lung cancer.

[85] Takimoto T, Kijima T, Otani Y, Nonen S, Namba Y, Mori M, Yokota S, Minami S, Komuta K, Uchida J, Imamura F, Furukawa M, Tsuruta N (2013). Polymorphisms of CYP2D6 gene and gefitinib-induced hepatotoxicity. Clinical lung cancer.

[86] Lee KW, Chan SL (2016). Hepatotoxicity of targeted therapy for cancer. Expert opinion on drug metabolism & toxicology.

[87] Liu C, Yang Z, Wang L, Lu Y, Tang B, Miao H, Xu Q, Chen X (2015). Combination of sorafenib and gadolinium chloride (GdCl3) attenuates dimethylnitrosamine(DMN)-induced liver fibrosis in rats. BMC gastroenterology.

[88] Yamamoto N, Yamasaki T, Takami T, Uchida K, Fujisawa K, Matsumoto T, Saeki I, Terai S, Sakaida I (2016). Deferasirox, an oral iron chelator, prevents hepatocarcinogenesis and adverse effects of sorafenib. Journal of clinical biochemistry and nutrition.

[89] Friedman SL (2008). Hepatic stellate cells: Protean, multifunctional, and enigmatic cells of the liver. Physiological Reviews.

[90] Schon HT, Bartneck M, Borkham-Kamphorst E, Natterman J, Lammers T, Tacke F, Weiskirchen R (2016). Pharmacological Intervention in Hepatic Stellate Cell Activation and Hepatic Fibrosis. Frontiers in pharmacology.

[91] Poelstra K, Prakash J, Beljaars L (2012). Drug targeting to the diseased liver. J Control Release.

[92] Wang LS, Chen YW, Li DG, Lu HM (2006). Arg-gly-asp-mannose-6-phosphate inhibits activation and proliferation of hepatic stellate cells in vitro. World journal of gastroenterology.

[93] van Beuge MM, Poelstra K, Prakash J (2012). Specific delivery of kinase inhibitors in nonmalignant and malignant diseases. Expert opinion on drug delivery.

[94] Wang HL, Thorling CA, Liang XW, Bridle KR, Grice JE, Zhu YA, Crawford DHG, Xu ZP, Liu X, Roberts MS (2015). Diagnostic imaging and therapeutic application of nanoparticles targeting the liver. Journal of Materials Chemistry B.

[95] Chai NL, Fu Q, Shi H, Cai CH, Wan J, Xu SP, Wu BY (2012). Oxymatrine liposome attenuates hepatic fibrosis via targeting hepatic stellate cells. World journal of gastroenterology.

